# Dynamics of the stochastic chemostat with Monod-Haldane response function

**DOI:** 10.1038/s41598-017-13294-3

**Published:** 2017-10-20

**Authors:** Liang Wang, Daqing Jiang, Gail S. K. Wolkowicz, Donal O’Regan

**Affiliations:** 1 School of Mathematics and Statistics, Northeast Normal University, Changchun, 130024 P. R. China; 2 Department of Mathematics and Statistics, McMaster University, Hamilton, ON L8S 4K1 Canada; 3 College of Science, China University of Petroleum (East China), Qingdao, 266580 P. R. China; 4 School of Mathematics, Statistics and Applied Mathematics, National University of Ireland, Galway, Ireland; 5 Nonlinear Analysis and Applied Mathematics (NAAM)-Research Group, King Abdulaziz University, Jeddah, Saudi Arabia

## Abstract

The stochastic chemostat model with Monod-Haldane response function is perturbed by environmental white noise. This model has a global positive solution. We demonstrate that there is a stationary distribution of the stochastic model and the system is ergodic under appropriate conditions, on the basis of Khasminskii’s theory on ergodicity. Sufficient criteria for extinction of the microbial population in the stochastic system are established. These conditions depend strongly on the Brownian motion. We find that even small scale white noise can promote the survival of microorganism populations, while large scale noise can lead to extinction. Numerical simulations are carried out to illustrate our theoretical results.

## Introduction

The chemostat, known as a continuous stir tank reactor (CSTR) in the engineering literature, is a basic piece of laboratory apparatus used for the continuous culture of microorganisms. It occupies a central place in mathematical ecology and has played an important role in many fields. In ecology it is often viewed as a model of simple lake or an ocean system. It can also model the wastewater treatment process^1^ or biological waste decomposition^2^. In some fermentation processes, the chemostat plays a central role in the commercial production of genetically altered organisms. The theoretical investigation of the dynamics of microbial interaction in the chemostat was initiated by Monod^3^ and Novick *et al*.^4^ in 1950. There is also an extensive literature on the chemostat (for example, see refs^5–11^ and the references therein for recent research) concerned with the dynamics of various types of chemostat models.

The classic chemostat model with single species and single limiting substrate takes the form

11 $$\{\begin{array}{ccc}\frac{dS(t)}{dt} & = & Q({S}^{0}-S(t))-\frac{1}{\delta }(S(t))x(t),\\ \frac{dx(t)}{dt} & = & \mu (S(t))x(t)-Qx(t),\end{array}$$

where *S*(*t*), *x*(*t*) stand for the concentrations of nutrient and microbial population at time t respectively; *S* ^0^ denotes the imput concentration of nutrient and *Q* represents the volumetric flow rate of the mixture of nutrient and microorganism; the coefficient *δ* is the ratio of the biomass of the microbial population produced by the nutrient consumed. The growth rate of the microbial population is represented by the function *μ*(*S*) ($\mu (S)\le m < \infty$), which is generally assumed to be non-negative. That is $\mu \mathrm{(0)}\,=\,0$ *, μ*(*S*) > 0 for *S* > 0.

Some experiments and observations indicate that not only insufficient nutrient but also excessive nutrient may inhibit the growth of a microbial population in the chemostat. To model such growth, Andrews^12^ suggested a non-monotonic response function:

1.2 $$\mu (S)=\frac{mS}{a+S+K{S}^{2}},$$

which is called the Monod-Haldane growth rate (inhibition rate). Parameter *m* is the maximum growth rate of the microorganism, *a* is the Michaelis-Menten (or half-saturation) constant. The term *KS* ^2^ models the inhibitory effect of the substrate at high concentrations. The chemostat model with Monod-Haldane response function has been studied by many researchers (see e.g. Wang *et al*.^13^, Pang *et al*.^14^, Baek *et al*.^15^ and the references there in). Taking the non-monotone functional response into account, the chemostat model (1.1) becomes:

1.3 $$\{\begin{array}{rcl}\frac{dS}{dt} & = & {Q}({S}^{0}-S)-\frac{1}{\delta }\frac{mS}{a+S+K{S}^{2}}x,\\ \frac{dx}{dt} & = & \frac{mS}{a+S+K{S}^{2}}x-Qx\mathrm{.}\end{array}$$

System (1.3) has a trivial equilibrium point (*S* ^0^, 0). It is an asymptotically stable steady node when ${Q} > \frac{m{S}^{0}}{a+{S}^{0}+K{S}^{{0}^{2}}}$ and a saddle point when ${Q} < \frac{m{S}^{0}}{a+{S}^{0}+K{S}^{{0}^{2}}}$. In addition, there are two interior equilibria $({S}_{1}^{\ast },{x}_{1}^{\ast })$, $({S}_{2}^{\ast },{x}_{2}^{\ast })$ and ${S}_{1}^{\ast }$, ${S}_{2}^{\ast }$ satisfy the equation

1.4 $$QK{S}^{2}+(Q-m)S+Qa=\mathrm{0,}\,Q < m\mathrm{.}$$

It is not difficult to verify that one of the two interior equilibria is a stable nodal point and the other is a saddle point. In mathematical biology, when the orbits tend to the stable nodal point (*S* ^*^, *x* ^*^), the continuous culture of microorganism is considered to be successful. We refer the reader to Chen^16^ for more details.

System (1.3) is deterministic with parameters assumed to be constant. Environmental fluctuations are ignored, which, from the biological point of view, is unrealistic. Ecosystem dynamics are inevitably affected by environmental noise and it is more realistic to include the effect of stochasticity rather than to study models that are entirely deterministic. With respect to chemostat models, even though the experimental results that are observed in well-controlled laboratory conditions have been shown to match closely with the prediction of deterministic models involving ordinary differential equations, we cannot ignore the difference that may occur in operational conditions. Imhof *et al*.^17^ derived a stochastic chemostat model by considering a discrete-time Markov process with jumps corresponding to the addition of a centered Gaussian term to the deterministic model. With the time step converging to zero, they reported that the stochastic model may lead to extinction in some cases in which the deterministic model predicts persistence. In^18^, Campillo *et al*. considered a set of stochastic chemostat models that are valid on different scales. Xu and Yuan^19^ dealt with the stochastic chemostat in which the maximal growth rate *m* is perturbed by white noise and obtained a new break-even concentration $\tilde{\lambda }$ which completely determines the persistence or extinction of the microbial population. Zhao and Yuan^20^ further computed the probability for extinction and persistence in mean of the microbial population in^19^ using stochastic calculus. Wang *et al*.^21^ investigated the periodic solutions for the stochastic chemostat model with periodic washout rate, on the basis of Khasminskii’s theory (see, ref.^22^ Chapter 3) for periodic Markov processes.

In this paper, the dynamical behaviour of a two-dimensional chemostat model with Monod-Haldane response function under stochastic perturbation is investigated. The white noise is incorporated in stochastic system (1.3) to model the effect of a randomly fluctuating environment. The substrate and microorganism population are usually estimated by an average value plus errors (i.e. noise intensities) which are usually normally distributed. The standard deviations of the errors may depend on the population sizes. Utilizing the approach used in^17^ to include stochastic effects (readers can also refer to^23^, Appendix A to see the construction of this kind of stochastic model), the stochastic chemostat model with Monod-Haldane response function takes the form:

1.5 $$\{\begin{array}{rcl}dS & = & [Q({S}^{0}-S)-\frac{1}{\delta }\frac{mS}{a+S+K{S}^{2}}x]\,dt+{\sigma }_{1}Sd{B}_{1}(t),\\ dx & = & [\frac{mS}{a+S+K{S}^{2}}x-Qx]\,dt+{\sigma }_{2}xd{B}_{2}(t),\end{array}$$

where *B*_1_(*t*), *B*_2_(*t*) are independent standard (i.e. with variance *t*) Brownian motion defined in a complete probability space $({\rm{\Omega }}, {\mathcal F} ,{\{{ {\mathcal F} }_{t}\}}_{t\ge 0},P)$, and ${\sigma }_{1}^{2}\mathrm{,\ }{\sigma }_{2}^{2} > 0$ are their intensities. Since an ODE model is never exact, but only an approximation of reality; adding a linear perturbation using Brownian motion (terms ${\sigma }_{1}S{\dot{B}}_{1}$, ${\sigma }_{2}x{\dot{B}}_{2}$) as in model (1.5) helps to determine how the dynamics can possibly change by approximating the error in the ODE model, and the error is normally distributed, ${\sigma }_{i}({\sigma }_{i}^{2} > \mathrm{0)}$, *i* = 1, 2 are constants that reflect the sizes of error (stochastic effects).

Very few other studies have appeared on the stochastic chemostat model with the Monod-Haldane functional response. Campillo *et al*.^24^ considered a stochastic model of the chemostat, with both non-inhibitory (Monod) and inhibitory (Haldane) growth functions, as a diffusion process and used a finite difference scheme to approximate the solutions of the associated Fokker-Planck equation. They considered the stochastic model from a ‘demographic noise’ point of view. Instead of using *S* and *x* in the stochastic terms in model (1.5), they used $\sqrt{S}$ and $\sqrt{x}$, respectively.

In this paper, our aim is to reveal how the environmental noise affects a microbial population in the chemostat with Monod-Haldane response function. First, in section 2 we prove that there is a unique positive solution for model (1.5). Then in section 3 we show that for any initial value $(S\mathrm{(0),}\,x\mathrm{(0))}\in {{\mathbb{R}}}_{+}^{2}$, there is a stationary distribution for system (1.5) and it is ergodic under appropriate conditions. Sufficient conditions for extinction of the microorganism are presented in section 4. Finally, to illustrate our main conclusions, examples and numerical simulations are given in section 5.

## Existence and uniqueness of the positive solution

Before investigating the dynamical behavior of system (1.5), the existence of a global positive solution is proved. In order to ensure the stochastic chemostat make sense, we need to show at least not only that this SDE model has a unique global solution but also that the solution will remain in ${{\mathbb{R}}}_{+}^{2}$ whenever it starts from there. First we give the definition of a solution of stochastic differential equation (see^25^, Chapter 2 for more details). Throughout this paper, unless otherwise specified, let $({\rm{\Omega }}, {\mathcal F} ,\{{ {\mathcal F} }_{t}{\}}_{t\ge 0},P)$ be a complete probability space with filtration ${\{{ {\mathcal F} }_{t}\}}_{t\ge 0}$ satisfying the usual conditions (i.e. it is right continuous and ${ {\mathcal F} }_{0}$ contains all *P*-null sets). Let ${{\mathbb{R}}}_{+}^{l}=\{x\in {{\mathbb{R}}}^{l}:{x}_{i} > 0\,for\,all\,1\le i\le l\}$ and $B(t)={({B}_{1}(t),\ldots ,{B}_{m}(t))}^{T}$, *t* ≥ 0 be an *m*-dimensional Brownian motion defined on the space. Let $X({t}_{0})={X}_{0}$ $\mathrm{(0}\le {t}_{0} < T < \infty )$ be an ${ {\mathcal F} }_{{t}_{0}}$-measurable ${{\mathbb{R}}}^{l}$-valued random variable such that ${\mathbb{E}}|{X}_{0}{|}^{2} < \infty$. Let *f*: ${{\mathbb{R}}}^{l}\times [{t}_{0},T]\to {{\mathbb{R}}}^{l}$ and *g*: ${{\mathbb{R}}}^{l}\times [{t}_{0},T]\to {{\mathbb{R}}}^{l\times m}$ be both Borel measurable. Consider the *l*-dimensional stochastic differential equation of It $\hat{o}$ type

2.1 $$dX(t)=f(X(t),t)dt+g(X(t),t)dB(t)\,\,on\,{t}_{0}\le t\le T$$

with initial value *X*(*t*_0_) = *X*_0_. SDE (2.1) is equivalent to the following stochastic integral equation:

2.2 $$X(t)={X}_{0}+{\int }_{{t}_{0}}^{t}f(X(s),s)ds+{\int }_{{t}_{0}}^{t}f(X(s),s)dB(s)\,on\,{t}_{0}\le t\le T$$

**Definition 2.1** An ${{\mathbb{R}}}^{l}$-valued stochastic process ${\{X(t)\}}_{{t}_{0}\le t\le T}$ is called a solution of equation (2.1) if it has the following properties:

- {*X*(*t*)} *is continuous and* ${ {\mathcal F} }_{t}$-*adapted*;
- $$f(X(t),t)\in { {\mathcal L} }^{1}([{t}_{0},T];{{\mathbb{R}}}^{l})$$ *and* $$g(X(t),t)\in { {\mathcal L} }^{2}([{t}_{0},T];{{\mathbb{R}}}^{l\times m})$$;
- equation (2.2) *holds for every* $t\in [{t}_{0},T]$ *with probability 1*.

A solution {*X*(*t*)} is said to be unique if any other solution $\{\bar{X}(t)\}$ is indistinguishable from {*X*(*t*)}, that is

$$P\{X(t)=\bar{X}(t)\,for\,all\,{t}_{0}\le t\le T\}=\mathrm{1,}$$

where *P* denotes the probability of an event.

By utilizing the methods described in^25^, the coefficients of the equations would be required to satisfy a local Lipschitz condition and a linear growth condition. However, the Haldane function $S\to \mu (S)=\frac{mS}{a+S+K{S}^{2}}$ is nonlinear, coefficients of system (1.5) do not satisfy the linear growth condition. Thus in this section, using the Lyapunov analysis method^26^, we prove that the solution of the system (1.5) is positive and global.

**Theorem 2.1** For given initial value $(S\mathrm{(0),}\,x\mathrm{(0))}\in {{\mathbb{R}}}_{+}^{2}$, there is a unique solution $(S(t),x(t))$ of system (1.5) defined for all t ≥ 0, and the solution remains in ${{\mathbb{R}}}_{+}^{2}$ with probability one, i.e. $(S(t),x(t))\in {{\mathbb{R}}}_{+}^{2}$ for t ≥ 0 almost surely.

**Proof:** Consider the diffusion process as follows

2.3 $$\{\begin{array}{rcl}d\mu  & = & [Q(\frac{{S}^{0}}{{e}^{\mu }}-1)-\frac{1}{\delta }\frac{m{e}^{\nu }}{a+{e}^{\mu }+K{e}^{2\mu }}-\frac{1}{2}{\sigma }_{1}^{2}]\,dt+{\sigma }_{1}d{B}_{1}(t),\\ d\nu  & = & [\frac{m{e}^{\mu }}{a+{e}^{\mu }+K{e}^{2\mu }}-Q-\frac{1}{2}{\sigma }_{2}^{2}]\,dt+{\sigma }_{2}d{B}_{2}(t\mathrm{).}\end{array}$$

Since the coefficients of system (2.3) are locally Lipschitz continuous, there is a unique local solution for system (2.3). Let *S* = *e* ^*μ*^, *x* = *e* ^*μ*^, Itô’s formula (given in Section 3) implies that system (1.5) has a unique local positive solution. Hence it suffices to prove that this unique local positive solution of system (1.5) is global.

On the basis of the discussion above, we know that there is a unique local solution (*S*(*t*), *x*(*t*)) on $t\in \mathrm{[0},{\tau }_{e})$, where *τ*_*e*_ is the blow up time. If we can prove *τ*_*e*_ = ∞ a.s., then the solution will be global. Choose ${n}_{0}\ge 0$ big enough in order for *S*(0), *x*(0) to lie within the interval $[\frac{1}{{n}_{0}},{n}_{0}]$. For each integer $n\ge {n}_{0}$, define the stopping time:

$${\tau }_{n}=inf\{t\in \mathrm{[0},{\tau }_{e}):\,\min \,\{S(t),x(t)\}\le \frac{1}{n}\,or\,{\rm{\max }}\,\{S(t),x(t)\}\ge n\}.$$

Throughout this paper, we set $in\,f\varphi =\infty$ (as usual, *ϕ* denotes the empty set). Clearly, *τ*_*n*_ is increasing as $n\to \infty$. Set ${\tau }_{\infty }=\mathop{lim}\limits_{n\to \infty }{\tau }_{n}$, where ${\tau }_{\infty }\le {\tau }_{e}$ a.s. If we prove that ${\tau }_{\infty }=\infty$ a.s., then ${\tau }_{e}=\infty$ and $(S(t),x(t))\in {{\mathbb{R}}}_{+}^{2}$ a.s. for all $t\ge 0$. In other words, to complete the proof all we need to show is that ${\tau }_{\infty }=\infty \,a\mathrm{.}s\mathrm{.}$ If not, there exists a pair of constants *T* > 0 and $\varepsilon \in \mathrm{(0},\mathrm{1)}$ such that

$$P\{{\tau }_{\infty }\le T\} > \varepsilon \mathrm{.}$$

Hence there is an integer *n*_1_ ≥ *n*_0_ such that

2.4 $$P\{{\tau }_{n}\le T\} > \varepsilon ,\,for\,all\,n\ge {n}_{1}\mathrm{.}$$

Define a *C* ^2^-function $V(S,x)$: ${{\mathbb{R}}}_{+}\times {{\mathbb{R}}}_{+}\to {\mathbb{R}}$ by

$$V(S,x)=S-A-A\,\mathrm{log}\,\frac{S}{A}+\frac{1}{\delta }(x-1-\,\mathrm{log}\,x),$$

where $A=\frac{Qa}{m}$. Obviously, this function is non-negative for all *S*, *x* ≥ 0. Using Itô’s formula (see Theorem 3.1), we get

$$\begin{array}{rcl}dV(S,x) & = & \frac{S-A}{S}[(Q({S}^{0}-S)-\frac{1}{\delta }\frac{mSx}{a+S+K{S}^{2}})dt+{\sigma }_{1}(S-A)d{B}_{1}(t)]+\frac{1}{2}{\sigma }_{1}^{2}Adt\\  &  & +\frac{1}{\delta }(x-\mathrm{1)}[(\frac{mSx}{a+S+K{S}^{2}}-Qx)dt+{\sigma }_{2}d{B}_{2}(t)]+\frac{1}{2\delta }{\sigma }_{2}^{2}dt\\  & := &  {\mathcal L} V(S,x)dt+{\sigma }_{1}(S-A)d{B}_{1}(t)+\frac{1}{\delta }{\sigma }_{2}(x-\mathrm{1)}d{B}_{2}(t),\end{array}$$

where ${\mathcal L}$ is the generating operator of system (1.5) and

$$\begin{array}{rcl} {\mathcal L} V(S,x) & = & Q(1-\frac{A}{S})({S}^{0}-S)-\frac{1}{\delta }\frac{mSx}{a+S+K{S}^{2}}+\frac{Amx}{a+S+K{S}^{2}}\\  &  & +\frac{x-1}{\delta }[\frac{mS}{a+S+K{S}^{2}}-Q]+\frac{1}{2}{\sigma }_{1}^{2}A+\frac{1}{2\delta }{\sigma }_{2}^{2}\\  &  & \le Q{S}^{0}+\frac{Am}{a\delta }-\frac{Q}{\delta }x+\frac{Q}{\delta }+\frac{1}{2}{\sigma }_{1}^{2}A+\frac{1}{2\delta }{\sigma }_{2}^{2}\\  & = & Q{S}^{0}+\frac{Q}{\delta }+\frac{Qa}{2m}{\sigma }_{1}^{2}+\frac{1}{2\delta }{\sigma }_{2}^{2}\\  & \triangleq  & {C}^{\ast },\end{array}$$

*C* ^*^ is a positive constant. Therefore

$$\begin{array}{c}{\int }_{0}^{{\tau }_{n}\wedge T}dV(S(t),x(t))\le {\int }_{0}^{{\tau }_{n}\wedge T}{C}^{\ast }\,dt+{\int }_{0}^{{\tau }_{n}\wedge T}{\sigma }_{1}(S-A)d{B}_{1}(t)\\ \quad \quad \quad \quad \quad \quad \quad \quad \quad \,\,+{\int }_{0}^{{\tau }_{n}\wedge T}\frac{1}{\delta }{\sigma }_{2}(x-\mathrm{1)}d{B}_{2}(t),\end{array}$$

Taking expectation of both sides implies that

25 $$\begin{array}{c}EV(S({\tau }_{n}\wedge T),x({\tau }_{n}\wedge T))\le V(S\mathrm{(0)},x\mathrm{(0))}+E{\int }_{0}^{{\tau }_{n}\wedge T}{C}^{\ast }\,dt\\ \quad \quad \quad \quad \quad \quad \quad \quad \,\,\,\quad \quad \le V(S\mathrm{(0)},x\mathrm{(0))}+{C}^{\ast }T\mathrm{.}\end{array}$$

Set ${{\rm{\Omega }}}_{n}={\tau }_{n}\le T$ for all *n* ≤ *n*1. Then by (2.4) $P({{\rm{\Omega }}}_{n})\ge \varepsilon$. Note that for every $\omega \in {{\rm{\Omega }}}_{n}$, there exists at least one $S({\tau }_{n},\omega )$ or $x({\tau }_{n},\omega )$ that equals either *n* or $\frac{1}{n}$, and then $V(S({\tau }_{n}),x({\tau }_{n}))$ is no less than

$$n-1-\,\mathrm{log}\,n\,or\,\frac{1}{n}-1-\,\mathrm{log}\,\frac{1}{n}=\frac{1}{n}-1+\,\mathrm{log}\,n\mathrm{.}$$

Consequently,

$$V(S({\tau }_{n}),x({\tau }_{n}))\ge (n-1-\,\mathrm{log}\,n)\wedge (\frac{1}{n}-1+\,\mathrm{log}\,n)\mathrm{.}$$

It then follows from (2.4) and (2.5) that

$$\begin{array}{ccc}V(S\mathrm{(0)},x\mathrm{(0))}+{C}^{\ast }T & \ge  & E[{{\bf{1}}}_{{\Omega }_{n}(\omega )}V(S({\tau }_{n}),x({\tau }_{n}))]\\  & \ge  & \varepsilon [(n-1-\,\mathrm{log}\,n)\wedge (\frac{1}{n}-1+\,\mathrm{log}\,n)],\end{array}$$

where ${{\bf{1}}}_{{{\rm{\Omega }}}_{n}(\omega )}$ is the indicator function of Ω_*n*_. Letting *n* → ∞ leads to the contradiction that $\infty  > V(S\mathrm{(0)},x\mathrm{(0))}$ $+{C}^{\ast }T=\infty$. Thus we have *τ*_∞_ = ∞ a.s.☐

## Stationary distribution and ergodicity

In the study of the dynamics for stochastic systems, ergodicity is one of the most important and significant characteristics. The ergodic property for chemostat implies that the stochastic model has a unique stationary distribution which predicts the survival of the microbial population in the future. In addition, the ergodic property gives a reason why the integral average of a population system converges to a fixed point whilst the population system may fluctuate around as time goes by. Thus research on the ergodicity of population system is essential from a biological perspective (see^27^). In this section, before the proof of the ergodicity, several auxiliary results are derived for the stationary distribution. For more details, we refer the reader to^28^. First we introduce the multi-dimensional Itô formula.

**Theorem 3.1** (*The multi-dimensional Itô formula) (Chapter 1)* ^25^ *Let X*(*t*) *be a l*-*dimensional It* $\hat{o}$ *process on t* ≥ 0 *with the stochastic differential*

$$dX(t)=f(t)dt+g(t)dB(t),$$

*where* $f\in { {\mathcal L} }^{1}({{\mathbb{R}}}_{+};{{\mathbb{R}}}^{d})$ *and* $g\in { {\mathcal L} }^{2}({{\mathbb{R}}}_{+};{{\mathbb{R}}}^{d\times m})$. *Let* $V\in {C}^{\mathrm{2,1}}({{\mathbb{R}}}^{d}\times {{\mathbb{R}}}_{+};{\mathbb{R}})$. *Then V*(*X*(*t*), *t*) *is an It* $\hat{o}$ *process with the stochastic differential given by*

3.1 $$\begin{array}{rcl}dV(X(t),t) & = & [{V}_{t}(X(t),t)+{V}_{X}(X(t),t)f(t)+\frac{1}{2}\,trace\,({g}^{T}(t){V}_{XX}(X(t),t)g(t))]dt\\  &  & +{V}_{X}(X(t),t)g(t)dB(t)\,\,\,a\mathrm{.}s\mathrm{.}\end{array}$$

Here ${V}_{t}=\frac{\partial V}{\partial t}$, ${V}_{X}=(\frac{\partial V}{\partial {X}_{1}},\ldots ,\frac{\partial V}{\partial {X}_{l}})$, ${V}_{XX}={(\frac{{\partial }^{2}V}{\partial {X}_{i}\partial {X}_{j}})}_{l\times l}$Assume *Y*(*t*) is a regular time-homogeneous Markov Process in ${{\mathbb{E}}}^{l}$ (${{\mathbb{E}}}^{l}$ denotes the *l*-dimensional Euclidean space) described by the stochastic differential equation

3.2 $$dY(t)=b(Y)dt+\sum _{r\mathrm{=1}}^{l}{g}_{r}(Y)d{B}_{r}(t),$$

Brownian motion *B*_*r*_, *r* = 1, …, *l* are independent. The diffusion matrix is

$${\rm{\Lambda }}(y)=({\lambda }_{ij}(y)),\,{\lambda }_{ij}(y)=\sum _{r\mathrm{=1}}^{l}{g}_{r}^{i}(y){g}_{r}^{j}(y\mathrm{).}$$

Define the differential operator ${\mathcal L}$ associated with equation (3.2) by

$$ {\mathcal L} =\sum _{k\mathrm{=1}}^{l}{b}_{k}(y)\frac{\partial }{\partial {y}_{k}}+\frac{1}{2}\sum _{k,j\mathrm{=1}}^{l}{\lambda }_{kj}(y)\frac{{\partial }^{2}}{\partial {y}_{k}\partial {y}_{j}}\mathrm{.}$$

**Lemma 3.1** (Rafail Khasminskii, 2011) Assume that there exists a bounded domain $U\subset {{\mathbb{E}}}^{l}$ with regular boundary Γ, having the following properties:

(***B.1***) *In the domain U and some neighborhood thereof, the smallest eigenvalue of the diffusion matrix* ${\rm{\Lambda }}(x)$ *is bounded away from zero*.

(***B.2***) *If* $x\in {{\mathbb{E}}}^{l}\backslash U$, *the mean time τ for the paths issuing from x to reach the set U is finite, and* $\mathop{{\rm{\sup }}\,}\limits_{x\in D}{E}_{x}\tau  < \infty$ *for every compact subset* $D\subset {{\mathbb{E}}}^{l}$. *Here E means expectation*.

*Then the Markov process X*(*t*) *has a stationary distribution* $\mu (\cdot )$. *Moreover, let f*(*x*) *be a function integrable with respect to the measure μ*. *Then the probability*

$$P\{\mathop{\mathrm{lim}}\limits_{T\to \infty }\frac{1}{T}{\int }_{0}^{T}f(X(t))dt={\int }_{{E}^{l}}f(x)\mu (dx)\}=\mathrm{1,}$$

*for all* $$x\in {{\mathbb{E}}}^{l}$$.

**Remark 3.1** *The proof is given in* ^22^. *To show the existence of the stationary distribution* $\mu (\cdot )$ *of system (1.5)*, *it is enough for us to take* ${{\mathbb{R}}}_{+}^{2}$ *as the whole space. To validate* (*B.1*), *we need to prove that for any bounded domain* $D\subset {{\mathbb{R}}}_{+}^{2}$, *there is a positive number M*_0_ *such that* $\sum _{i,j\mathrm{=1}}^{2}{\lambda }_{ij}(x){\xi }_{i}{\xi }_{j}\ge {M}_{0}|\xi {|}^{2}\mathrm{,\ }x\in \bar{D}\mathrm{,\ }\xi \in {{\mathbb{R}}}_{+}^{2}$ (*see* ^29^ *Chapter 3*, *p.103 and Rayleigh’s principle in* ^30^ *Chapter 6*, *p.349*). *To validate* (*B.2*), *it is sufficient to show that there exists a neighborhood U and a non-negative C* ^2^-*function V*(*S*, *x*) *such that for any* ${{\mathbb{E}}}^{l}\backslash U$, ${\mathcal L} V(S,x)$ *is negative (for more details see* ^31^, *p.1163*).

**Definition 3.1** (Regularity, Recurrence) A Markov process X(t) is said to be regular, if for any $0 < T < \infty$,

$$P\{\mathop{sup}\limits_{0\le t\le T}|X(t)|=\infty \}=0.$$

*A regular process X*(*t*) *described by (3.2) with nonsingular diffusion matrix (i.e*., *the smallest eigenvalue of* Λ(*x*) *is bounded away from zero in every bounded domain in* ${{\mathbb{E}}}^{l}$.) *is said to be recurrent if there exists a bounded domain U such that for all* $x\in {{\mathbb{E}}}^{l}\backslash U$,

$$P\{{\tau }_{x} < \infty \}=\mathrm{1,}$$

*where* ${\tau }_{x}={\rm{\inf }}\{t > \mathrm{0:\ }X\mathrm{(0)}=x,\,X(t)\in U\}$ *is the hitting time of U* for *X*(*t*).

**Remark 3.2** *The definition of regularity and recurrence come from* ^32^ *. Let X*(*t*) *be a regular temporally homogeneous Markov process in* ${{\mathbb{E}}}^{l}$. *If X*(*t*) *is recurrent with respect to some bounded domain U*, *then it is recurrent with respect to any nonempty domain in* ${{\mathbb{E}}}^{l}$. *Since the existence of the positive solution for model (1.5) has been obtained by Theorem 2.1*, *it is enough for us to take* ${{\mathbb{R}}}_{+}^{2}$ *as the whole space*.

Before proving the ergodicity, we define the notation ${f}^{u}=\mathop{{\rm{\sup }}}\limits_{t\in \mathrm{[0,}\infty )}f(t)$, ${f}^{l}=\mathop{{\rm{\inf }}}\limits_{t\in \mathrm{[0,}\infty )}f(t)$. Define a continuous function $G:{{\mathbb{R}}}_{+}\to {\mathbb{R}}$ by

$$\begin{array}{ccc}G(S) & = & -(Q-{\sigma }_{1}\mathop{Q}\limits^{ \sim }K)(S-{S}^{0}{)}^{2}+{\sigma }_{1}(\mathop{Q}\limits^{ \sim }-m+2\mathop{Q}\limits^{ \sim }K{S}^{0})(S-{S}^{0})\\  &  & -{\sigma }_{1}[m{S}^{0}-\mathop{Q}\limits^{ \sim }(a+{S}^{0}+K{{S}^{0}}^{2})].\end{array}$$

where $$\tilde{{Q}}=Q+\frac{1}{2}{\sigma }_{2}^{2}$$.

**Theorem 3.2** *If σ*_1_, *σ*_2_ *satisfy*:

3.3 $${\sigma }_{1} < {G}_{0},\,{\sigma }_{1}^{2} < \frac{-2C}{{S}^{0}}\wedge Q$$

3.4 $$\mathop{Q}\limits^{ \sim } < \frac{m{S}^{0}}{a+{S}^{0}+K{{S}^{0}}^{2}},\,{\sigma }_{2}^{2} < 2Q,$$

*where* $C=\mathop{{\rm{\sup }}}\limits_{S\in \mathrm{(0,}\infty )}\frac{G(S)}{a+S+K{S}^{2}} < 0$, ${G}_{0}=\frac{Q}{\mathop{Q}\limits^{ \sim }K}\wedge \frac{4Q[m{S}^{0}-\mathop{Q}\limits^{ \sim }(a+{S}^{0}+K{S}^{02})]}{{(\mathop{Q}\limits^{ \sim }-m+2\mathop{Q}\limits^{ \sim }K{S}^{0})}^{2}+4\mathop{Q}\limits^{ \sim }K[m{S}^{0}-\mathop{Q}\limits^{ \sim }(a+{S}^{0}+K{{S}^{0}}^{2})]}$ *(symbol* $\wedge$ *means choose the smaller one)*. *Then for any initial value* $(S\mathrm{(0)},x\mathrm{(0))}\in {{\mathbb{R}}}_{+}^{2}$, *there is a stationary distribution* $\mu (\cdot )$ *for system (1.5) and the system is ergodic*.

**Proof:** For simplicity let *S*, *x* stand for *S*(*t*), *x*(*t*), respectively. We will verify that (B.1) and (B.2) hold under condition (3.3–3.4), according to Lemma 3.1 on the existence of the stationary distribution. System (1.5) can be written as the following system:

$$\begin{array}{ccc}d(\begin{array}{c}S\\ x\end{array}) & =(\begin{array}{c}Q({S}^{0}-S)-\frac{1}{\delta }\frac{mS}{a+S+K{S}^{2}}x\\ \frac{mS}{a+S+K{S}^{2}}x-Qx\end{array})dt & +(\begin{array}{c}{\sigma }_{1}S\\ 0\end{array})d{B}_{1}(t)\\  &  & +(\begin{array}{c}0\\ {\sigma }_{2}x\end{array})d{B}_{2}(t),\end{array}$$

hence the diffusion matrix is

$${\rm{\Lambda }}(S,x)=(\begin{array}{ll}{\sigma }_{1}^{2}{S}^{2} & 0\\ 0 & {\sigma }_{2}^{2}{x}^{2}\end{array})\mathrm{.}$$

Let *D* to be any bounded domain in ${{\mathbb{R}}}_{+}^{2}$, then there exists a positive constant ${M}_{0}=\,{\rm{\min }}\,\{{\sigma }_{1}^{2}{S}^{2}$, ${\sigma }_{2}^{2}{x}^{2},(S,x)\in \bar{D}\}$ such that

$$\sum _{i,j\mathrm{=1}}^{2}{\lambda }_{ij}(S,x){\xi }_{i}{\xi }_{j}={\sigma }_{1}^{2}{S}^{2}{\xi }_{1}^{2}+{\sigma }_{2}^{2}{x}^{2}{\xi }_{2}^{2}\ge {M}_{0}|\xi {|}^{2},\,for\,all\,(S,x)\in \bar{D},\xi \in \,{{\rm{R}}}^{2}\mathrm{.}$$

This implies that condition (B.1) is satisfied.

Next we will construct a nonnegative *C* ^2^-function *V*(*S*, *x*) and a closed set $U\in {{\mathbb{R}}}_{+}^{2}$ such that

$$\mathop{{\rm{\sup }}}\limits_{(S,x)\in {{\mathbb{R}}}_{+}^{2}\backslash U} {\mathcal L} V(S,x) < -R < \mathrm{0,}$$

where *R* is a positive constant. This assures that condition (B.2) is satisfied.

Choose a positive constant *M* big enough such that

(A) ${{\rm{\Phi }}}^{u}-M\lambda \le -2\,,\,$where $\lambda =-C-\frac{{S}^{0}}{2}{\sigma }_{1}^{2} > 0$ and the function ${\rm{\Phi }}(S)$ will be given later in (**B**). Define a *C* ^2^-function:

$$H(S,x)=M[S-{S}^{0}-{S}^{0}\,\mathrm{log}\,\frac{S}{{S}^{0}}-{\sigma }_{1}\,\mathrm{log}\,x]+\frac{1}{2Q}{(S-{S}^{0}+\frac{1}{\delta }x)}^{2}-\,\mathrm{log}\,S\mathrm{.}$$

From the partial derivative equation of *H*(*S*, *x*) we have

3.5 $$-\frac{1-MS+M{S}^{0}}{\delta S}+\frac{1}{Q\delta }(S-{S}^{0}+\frac{M{\sigma }_{1}S}{1-MS+M{S}^{0}})=0.$$

We derive the unique solution *S*_0_ of (3.5) from the monotonicity property of the left part. Thus the following equation

$$\frac{x}{M{\sigma }_{1}\delta }=\frac{S}{1-MS+M{S}^{0}}$$

has a unique solution (*S*_0_, *x*_0_) which is the minimum point of function *H*(*S*, *x*). Here ${x}_{0}=\frac{M{\sigma }_{1}\delta {S}_{0}}{1-M{S}_{0}+M{S}^{0}}$. Therefore $H(S,x)-H({S}_{0},{x}_{0})\ge 0$.

Next, we define a nonnegative *C* ^2^-function $V(\cdot ,\cdot )$: ${{\mathbb{R}}}_{+}^{2}\to {{\mathbb{R}}}_{+}$ by

$$V(S,x)=M[S-{S}^{0}-{S}^{0}\,\mathrm{log}\,\frac{S}{{S}^{0}}-{\sigma }_{1}\,\mathrm{log}\,x]+\frac{1}{2Q}{(S-{S}^{0}+\frac{1}{\delta }x)}^{2}-\mathrm{log}\,S-H({S}_{0},{x}_{0}\mathrm{).}$$

Denote $${V}_{1}(S,x)=S-{S}^{0}-{S}^{0}\,\mathrm{log}\,\frac{S}{{S}^{0}}$$, $${V}_{2}(S,x)={V}_{1}(S,x)-{\sigma }_{1}\,\mathrm{log}\,x$$, $${V}_{3}(S,x)=\frac{1}{2Q}{(S-{S}^{0}+\frac{1}{\delta }x)}^{2}$$.

Hence

3.6 $$ {\mathcal L} V(S,x)=M {\mathcal L} {V}_{2}+ {\mathcal L} {V}_{3}+ {\mathcal L} (-\mathrm{log}\,S\mathrm{).}$$

By Itô’s formula, we obtain that

$$\begin{array}{rcl} {\mathcal L} {V}_{1}(S,x) & = & \frac{S-{S}^{0}}{S}[Q({S}^{0}-S)-\frac{1}{\delta }\frac{mSx}{a+S+K{S}^{2}}]+\frac{{S}^{0}}{2}{\sigma }_{1}^{2}\\  &  & \le -\frac{Q{({S}^{0}-S)}^{2}}{S}+\frac{1}{\delta }\frac{m{S}^{0}x}{a+S+K{S}^{2}}+\frac{{S}^{0}}{2}{\sigma }_{1}^{2}\\  &  & \le -\frac{Q{({S}^{0}-S)}^{2}}{S}+\frac{m{S}^{0}}{a\delta }x+\frac{{S}^{0}}{2}{\sigma }_{1}^{2}\\  &  & \le -\frac{Q{({S}^{0}-S)}^{2}}{a+S+K{S}^{2}}+\frac{m{S}^{0}}{a\delta }x+\frac{{S}^{0}}{2}{\sigma }_{1}^{2},\end{array}$$

and

$$\begin{array}{ccc}{\mathscr{L}}(-{\rm{l}}{\rm{o}}{\rm{g}}\,x) & = & \mathop{Q}\limits^{ \sim }-\frac{mS}{a+S+K{S}^{2}}\\  & = & \frac{\mathop{Q}\limits^{ \sim }a+\mathop{Q}\limits^{ \sim }S+\mathop{Q}\limits^{ \sim }K{S}^{2}-mS}{a+S+K{S}^{2}}\\  & = & \frac{\mathop{Q}\limits^{ \sim }K{(S-{S}^{0})}^{2}+(\mathop{Q}\limits^{ \sim }-m+2\mathop{Q}\limits^{ \sim }K{S}^{0})(S-{S}^{0})-[mS-\mathop{Q}\limits^{ \sim }(a+{S}^{0}+K{{S}^{0}}^{2})]}{a+S+K{S}^{2}}.\end{array}$$

Thus

$$\begin{array}{ccc}{\mathscr{L}}{V}_{2}(S,x) & \le  & {\textstyle \tfrac{{\sigma }_{1}\mathop{Q}\limits^{ \sim }K{(S-{S}^{0})}^{2}+{\sigma }_{1}(\mathop{Q}\limits^{ \sim }-m+2\mathop{Q}\limits^{ \sim }K{S}^{0})(S-{S}^{0})-{\sigma }_{1}[mS-\mathop{Q}\limits^{ \sim }(a+{S}^{0}+K{{S}^{0}}^{2})]}{a+S+K{S}^{2}}}\\  &  & -\frac{Q{({S}^{0}-S)}^{2}}{a+S+K{S}^{2}}+\frac{m{S}^{0}}{a\delta }x+\frac{{S}^{0}}{2}{\sigma }_{1}^{2}\\  & = & {\textstyle \tfrac{-(Q-{\sigma }_{1}\mathop{Q}\limits^{ \sim }K)(S-{S}^{0}{)}^{2}+{\sigma }_{1}(\mathop{Q}\limits^{ \sim }-m+2\mathop{Q}\limits^{ \sim }K{S}^{0})(S-{S}^{0})-{\sigma }_{1}[mS-\mathop{Q}\limits^{ \sim }(a+{S}^{0}+K{{S}^{0}}^{2})]}{a+S+K{S}^{2}}}\\  &  & +\frac{m{S}^{0}}{a\delta }x+\frac{{S}^{0}}{2}{\sigma }_{1}^{2}\\  & = & \frac{G(S)}{a+S+K{S}^{2}}+\frac{m{S}^{0}}{a\delta }x+\frac{{S}^{0}}{2}{\sigma }_{1}^{2}.\end{array}$$

It follows from the condition (3.3) that the discriminant of function *G*(*S*) is negative and $Q-{\sigma }_{1}\tilde{Q}K > 0$. Then for any $S\in \mathrm{(0},\infty )$, function *G*(*S*) < 0. Take $C=\mathop{{\rm{\sup }}}\limits_{S\in (,\infty )}\frac{G(S)}{a+S+K{S}^{2}} < 0$, and let $\lambda =-C-\frac{{S}^{0}}{2}{\sigma }_{1}^{2} > 0$, then we get

3.7 $$ {\mathcal L} {V}_{2}(S,x)\le -\lambda +\frac{m{S}^{0}}{a\delta }x\mathrm{.}$$

In addition, since ${S}^{2}\le 2(S-{S}^{0}{)}^{2}+2{{S}^{0}}^{2}$, we have

38 $$\begin{array}{ccc}{\mathscr{L}}{V}_{3}(S,x) & = & -{(S-{S}^{0}+\frac{1}{\delta }x)}^{2}+\frac{{\sigma }_{1}^{2}}{2Q}{S}^{2}+\frac{{\sigma }_{2}^{2}}{2Q{\delta }^{2}}{x}^{2}\\  & = & -{(S-{S}^{0})}^{2}-\frac{1}{{\delta }^{2}}{x}^{2}-\frac{2}{\delta }(S-{S}^{0})x+\frac{{\sigma }_{1}^{2}}{2Q}{S}^{2}+\frac{{\sigma }_{2}^{2}}{2Q{\delta }^{2}}{x}^{2}\\  &  & \le -(1-\frac{{\sigma }_{1}^{2}}{Q}){(S-{S}^{0})}^{2}-\frac{1}{{\delta }^{2}}(1-\frac{{\sigma }_{2}^{2}}{2Q}){x}^{2}+\frac{2{S}^{0}}{\delta }x+\frac{{{S}^{0}}^{2}}{Q}{\sigma }_{1}^{2},\quad \quad \quad \quad \end{array}$$

and

39 $$\begin{array}{rcl} {\mathcal L} (-{\rm{l}}{\rm{o}}{\rm{g}}{\rm{S}}) & = & -\frac{Q{S}^{0}}{S}+Q+\frac{1}{\delta }\frac{mx}{a+S+K{S}^{2}}+\frac{1}{2}{\sigma }_{1}^{2}\\  &  & \le -\frac{Q{S}^{0}}{S}+Q+\frac{m}{a\delta }x+\frac{1}{2}{\sigma }_{1}^{2}\mathrm{.}\end{array}$$

Substituting (3.7–3.9) into (3.6),

$$\begin{array}{c}{\mathscr{L}}V(S,x)\le M(-\lambda +\frac{m{S}^{0}}{a\delta }x)-(1-\frac{{\sigma }_{1}^{2}}{Q}){(S-{S}^{0})}^{2}-\frac{1}{{\delta }^{2}}(1-\frac{{\sigma }_{2}^{2}}{2Q}){x}^{2}\\ \quad \quad \quad \quad \quad +\frac{2{S}^{0}}{\delta }x-\frac{Q{S}^{0}}{S}+Q+\frac{m}{a\delta }x+\frac{1}{2}{\sigma }_{1}^{2}+\frac{{{S}^{0}}^{2}}{Q}{\sigma }_{1}^{2}\\ \quad \quad \quad \quad =\,{\rm{\Phi }}(S)+{\rm{\Psi }}(x),\end{array}$$

where

310 $$(B)\{\begin{array}{ccc}{\rm{\Phi }}(S) & = & -(1-\frac{{\sigma }_{1}^{2}}{Q}){(S-{S}^{0})}^{2}-\frac{Q{S}^{0}}{S}+Q+\frac{1}{2}{\sigma }_{1}^{2}+\frac{{{S}^{0}}^{2}}{Q}{\sigma }_{1}^{2}\\ {\rm{\Psi }}(x) & = & -\frac{1}{{\delta }^{2}}(1-\frac{{\sigma }_{2}^{2}}{2Q}){x}^{2}+\frac{2{S}^{0}}{\delta }x+\frac{m}{a\delta }x+M(-\lambda +\frac{m{S}^{0}}{a\delta }x).\end{array}$$

In view of (3.3) and (3.4), we observe that

$$\begin{array}{c}{\rm{\Phi }}(S)+{{\rm{\Psi }}}^{u}\to -\infty ,\,{\rm{as}}\,S\to +\infty ,\\ {{\rm{\Phi }}}^{u}+{\rm{\Psi }}(x)\to -\infty ,\,{\rm{as}}\,x\to +\infty ,\\ {\rm{\Phi }}(S)+{{\rm{\Psi }}}^{u}\to -\infty ,\,{\rm{as}}\,S\to {0}^{+}\mathrm{.}\end{array}$$

The above cases lead to ${\mathcal L} V < -1$, respectively. Moreover, by reviewing condition (**A**) we obtain

$${{\rm{\Phi }}}^{u}+{\rm{\Psi }}(x)\to {{\rm{\Phi }}}^{u}-M\lambda \le -\mathrm{2,}\,{\rm{as}}\,x\to {0}^{+}\mathrm{.}$$

Take *ε* small enough, and let $U=[\varepsilon ,\frac{1}{\varepsilon }]\times [\varepsilon ,\frac{1}{\varepsilon }]$. Therefore

$$ {\mathcal L} V(S,x) < -1,\,(S,x)\in {U}^{c}\mathrm{.}$$

According to Remark 3.1, the conditions in Lemma 3.1 are satisfied. Thus stochastic chemostat model (1.5) has a stationary distribution and it is ergodic.☐

## Extinction

In this section, we discuss conditions that predict the failure of the continuous culture of microorganisms both in the case of environmental noise is ignored and in the case of big intensities of white noises, i.e. noise may lead to extinction of the microorganism in the reactor.

**Theorem 4.1** Let $\tilde{Q}=Q+\frac{1}{2}{\sigma }_{2}^{2}$. If one of the following conditions holds

(**i**) $$\tilde{Q}\ge m$$,

(**ii**) $$\tilde{Q} < m$$, *and* $$\mathrm{(1}-4aK){\tilde{Q}}^{2}-2\tilde{Q}m+{m}^{2} < 0$$.

*Then for any given initial value* $(S\mathrm{(0)},x\mathrm{(0))}\in {{\mathbb{R}}}_{+}^{2}$, *the solution* $(S(t),x(t))$ *of system (*1.5*) satisfies*

4.1 $$\mathop{\mathrm{lim}\,{\rm{\sup }}}\limits_{t\to \infty }\frac{1}{t}\,\mathrm{log}(x(t)) < 0\,a\mathrm{.}s\mathrm{.,}$$

*which means x*(*t*) *tends to zero exponentially almost surely. In other words*, *the microorganisms die out with probability one*.

**Proof:** By Itô’s formula, we have

$$\mathrm{log}\,x(t)=\,\mathrm{log}\,{x}_{0}+{\int }_{0}^{t}[\frac{mS}{a+S+K{S}^{2}}-Q-\frac{1}{2}{\sigma }^{2}]dt+{\sigma }_{2}{B}_{2}(t),$$

and this implies that

$$\mathop{\mathrm{lim}\,{\rm{\sup }}}\limits_{t\to \infty }\frac{1}{t}\,\mathrm{log}(x(t))=\mathop{\mathrm{lim}\,{\rm{\sup }}}\limits_{t\to \infty }\frac{1}{t}{\int }_{0}^{t}[\frac{mS(r)}{a+S(r)+K{S}^{2}(r)}-Q-\frac{1}{2}{\sigma }^{2}]dr\,a\mathrm{.}s\mathrm{..}$$

Denote $g(S)=\frac{mS}{a+S+K{S}^{2}}-\tilde{Q}$. If (**i**) holds, then $g(S)=\frac{-\tilde{Q}K{S}^{2}-(\tilde{Q}-m)S-\tilde{Q}a}{a+S+K{S}^{2}} < -\tilde{Q} < 0$; if (**ii**) holds, we obtain $g(S)\le \mathop{{\rm{\sup }}}\limits_{S\in \mathrm{(0,}\infty )}g(S) < 0$. Thus we conclude that

4.2 $$\mathop{\mathrm{lim}\,{\rm{\sup }}}\limits_{t\to \infty }\frac{1}{t}\,\mathrm{log}(x(t)) < \mathrm{0,}$$

that is, *x*(*t*) tends to zero exponentially with probability one,

$$\mathop{\mathrm{lim}}\limits_{t\to \infty }x(t)=0\,a\mathrm{.}s\mathrm{.}$$

The proof is complete.☐

**Remark 4.1** We refer to the condition (**ii**) in Theorem 4.1, which tells us that the microorganism species may die out when dilution rate Q and white noise are not large. While if the strength of white noise is large enough such that (i) holds, then the microorganism population will also become extinct, which never happens in the deterministic system (1.3) without environmental perturbations. Moreover, if the dilution rate Q is big enough which leads to the extinction of the species in the deterministic chemostat while the noise is not big (condition (i)), the microorganism species in stochastic chemostat (1.5) will also die out.

*From Theorem 4.1*, *under either condition (* ***i*** *) or (* ***ii*** *)*, *we obtain that there is some constant* $\lambda  > 0$ *such that*

$$\mathop{\mathrm{lim}\,{\rm{\sup }}}\limits_{t\to \infty }\frac{1}{t}\,\mathrm{log}(x(t))\le -\lambda \mathrm{.}$$

*That is to say*, *for a arbitrary small constant* $0 < \varepsilon  < \,{\rm{\min }}\{\frac{1}{2},\frac{\lambda }{2}\}$, *there exists a positive constant* ${T}_{1}={T}_{1}(\omega )$ *and a set* ${{\rm{\Omega }}}_{\varepsilon }$ *such that* $P\{{{\rm{\Omega }}}_{\varepsilon }\}\ge 1-\varepsilon$ *and* $\mathrm{log}\,x(t)\le -\frac{\lambda }{2}$ *for* $t\ge {T}_{1}$, $\omega \in {{\rm{\Omega }}}_{\varepsilon }$ *. Then* $x(t)\le {e}^{\frac{-\lambda t}{2}}$ *. Thus*

$$\mathop{\mathrm{lim}\,{\rm{\sup }}}\limits_{t\to \infty }x(t)\le \mathrm{0,\ \ }a\mathrm{.}s\mathrm{.,}$$

*which together with the positive property of the solution of system (*1.5*) implies that*

$$\mathop{\mathrm{lim}}\limits_{t\to \infty }x(t)=\mathrm{0,\ \ }a\mathrm{.}s\mathrm{.}$$

*Therefore the microorganism species x will go to extinction exponentially almost surely. In other words*, *the microorganism population will die out at an exponential rate with probability one*.

## Examples and numerical simulations

Generally, nonlinear stochastic differential equations, such as system (1.5), are usually too complex to be solved exactly, we can only theoretically prove the existence and uniqueness of the positive solution (see Theorem 2.1). In order to illustrate the analytical results in Section 3 and 4, we need to obtain approximate solutions of stochastic system (1.5) with given initial values and parameter values, via numerical methods and algorithms that can be implemented by Matlab. Due to Brownian motion and noise terms ${\sigma }_{1}Sd{B}_{1}(t)$, ${\sigma }_{2}xd{B}_{2}(t)$, we use Milstein’s higher order method^33^ to obtain the approximate solutions of stochastic chemostat model (1.5). J. Higham has showed the convergence of Milstein’s numerical method for stochastic differential equations (see Section 6, ref.^33^). We consider the following discretization equation in Milstein’s type to iteratively calculate the approximate solutions of stochastic system (1.5) in Matlab programs:

5.1 $$\{\begin{array}{rcl}{S}_{i+1} & = & {S}_{i}+[Q({S}^{0}-{S}_{i})-\frac{1}{\delta }\frac{m{S}_{i}{x}_{i}}{a+{S}_{i}+K{S}_{i}^{2}}]{\rm{\Delta }}t\\  &  & +{\sigma }_{1}{S}_{i}{\xi }_{\mathrm{1,}i}\sqrt{{\rm{\Delta }}t}+\frac{{\sigma }_{1}^{2}}{2}{S}_{i}({\xi }_{\mathrm{1,}i}^{2}{\rm{\Delta }}t-{\rm{\Delta }}t),\\ {x}_{i+1} & = & {x}_{i}+(\frac{m{S}_{i}{x}_{i}}{a+{S}_{i}+K{S}_{i}^{2}}-Q{x}_{i}){\rm{\Delta }}t\\  &  & +{\sigma }_{2}{x}_{i}{\xi }_{\mathrm{2,}i}\sqrt{{\rm{\Delta }}t}+\frac{{\sigma }_{2}^{2}}{2}{x}_{i}({\xi }_{\mathrm{2,}i}^{2}{\rm{\Delta }}t-{\rm{\Delta }}t),\end{array}$$

where ${\xi }_{\mathrm{1,}i}$ and ${\xi }_{\mathrm{2,}i}$ are $N\mathrm{(0,}\,\mathrm{1)}$-distributed independent Gaussian random variables, ${\sigma }_{1},{\sigma }_{2}$ are intensities of white noise and time increment ${\rm{\Delta }}t > 0$. In this way, the approximate solutions (performance results of (5.1) from Matlab) will converge to the explicit solutions of stochastic system (1.5) very fast.

**Figure 1:**
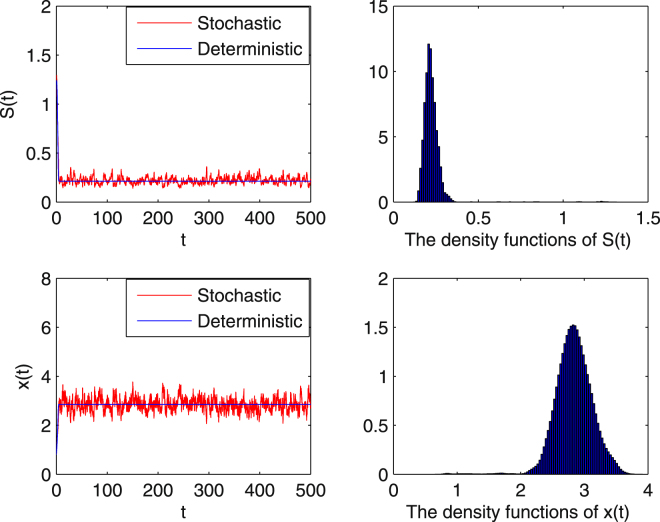
Numerical simulations of the solutions for system (1.5) and the corresponding deterministic system (1.3) with main parameter *Q* = 1. Both *S*(*t*) and *x*(*t*) are stationary Markov processes. The subgraphs on the right are the density functions of the correspondng stationary distributions for low level intensity Brownian motion with ${\sigma }_{1}=0.05,\,{\sigma }_{2}=0.1$.

**Example 5.1** From Theorem 3.2, we expect that under some appropriate conditions, the stochastic system (1.5) has a stationary distribution. Choose the initial value $(S\mathrm{(0)},x\mathrm{(0))}=\mathrm{(1.2},\mathrm{0.8)}$ and assume that the parameters in (1.5) are given by $Q=1$, $m=2$, ${S}^{0}=2$, $a=0.2$, $K=0.3$, $\delta =1.6$, and choose ${\sigma }_{1}=0.05$, ${\sigma }_{2}=0.1$. Hence conditions:

$$0.05={\sigma }_{1} < {G}_{0}=3.1192,\,0.0025={\sigma }_{1}^{2} < -\frac{2C}{{S}^{0}}\wedge Q=\mathrm{0.0086,}$$

$$1.0050=\mathop{Q}\limits^{ \sim } < \frac{m{S}^{0}}{a+{S}^{0}+K{{S}^{0}}^{2}}=1.1765,\,0.01={\sigma }_{2}^{2} < 2Q=2,$$

in Theorem 3.2 are all satisfied. Then we conclude that there is a stationary distribution (see the right two subgraphs in Fig. 1) of the stochastic system (1.5) and it is ergodic. Numerical simulations in Fig. 1 support this conclusion clearly, illustrating that the standard deviation *σ* keeps processes *S*(*t*), *x*(*t*) moving around the solution of the corresponding deterministic chemostat model.

**Figure 2:**
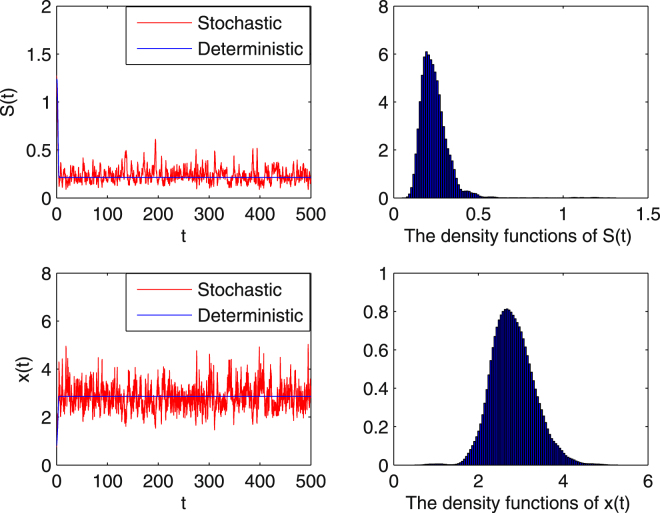
Numerical simulations of the solutions for system (1.5) and the corresponding deterministic system (1.3). The intensity of the Brownian motion is increased to ${\sigma }_{1}=0.1,\,{\sigma }_{2}=0.2$. The stochastic chemostat model still has a stationary distribution, even though the amplitude oscillations are stronger.

**Example 5.2** To further illustrate the effect of the Brownian motion *σ* on the stochastic chemostat model, we keep all the parameters in Example 5.1 unchanged but increase intensities to ${\sigma }_{1}=0.1$, and ${\sigma }_{2}=0.2$. We compute

$$0.1={\sigma }_{1} < {G}_{0}=\mathrm{2.9942,}\,0.01={\sigma }_{1}^{2} < -\frac{2C}{{S}^{0}}\wedge Q=\mathrm{0.0156,}$$

$$1.0200=\mathop{Q}\limits^{ \sim } < \frac{m{S}^{0}}{a+{S}^{0}+K{{S}^{0}}^{2}}=1.1765,0.04={\sigma }_{2}^{2}\, < \,2Q=2,$$

which means these parameter values satisfy the ergodic conditions (3.3–3.4). Figure 2 illustrates that the white noise still keeps processes $S(t)$, $x(t)$ moving around the solution of the deterministic chemostat model, while the random oscillations become stronger.

**Example 5.3** In order to show how the dilution rate and the random perturbation influence the extinction of the microbial population in system (1.5), we choose the initial value $(S\mathrm{(0)},x\mathrm{(0))}=\mathrm{(3},\mathrm{0.5)}$ and assume that $Q=2.2$, ${\sigma }_{1}=0.3$, ${\sigma }_{2}=0.4$ with the other parameters unchanged. Thus

$$2.280=Q+\frac{1}{2}{\sigma }_{2}^{2}=\tilde{Q} > m=2.$$

**Figure 3:**
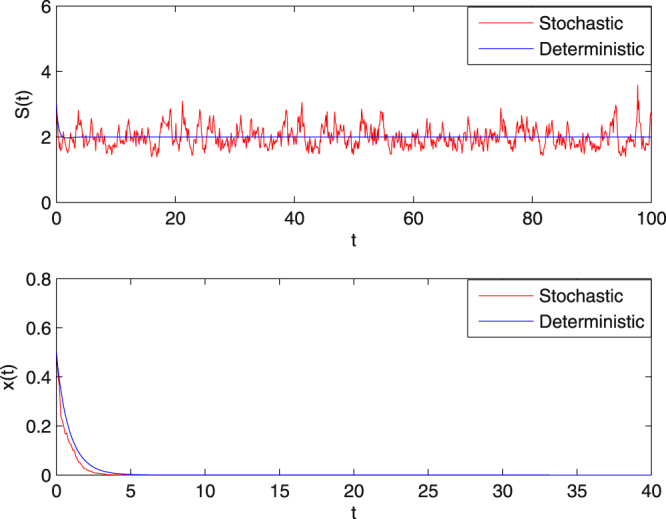
The sample paths of *S*(*t*) and *x*(*t*) for system (1.5) and the corresponding deterministic system (1.3) with *Q* = 2.2. The intensity of Brownian motion is chosen to be ${\sigma }_{1}=0.3,\,{\sigma }_{2}=0.4$. In the second subgraph, the large washout rate leads to the extinction of *x*(*t*) both in the deterministic and stochastic chemostat models.

We therefore conclude that by Theorem 4.1 (**i**), the solution of (1.5) obeys

$$\mathop{\mathrm{lim}\,{\rm{\sup }}}\limits_{t\to \infty }\frac{1}{t}\,\mathrm{log}(x(t)) < -\tilde{Q} < 0\,a\mathrm{.}s\mathrm{.,}$$

that is, *x*(*t*) tends to zero exponentially with probability one. On the other hand, for the corresponding deterministic chemostat model (1.3), condition

$$2.2=Q > \frac{m{S}^{0}}{a+{S}^{0}+K{{S}^{0}}^{2}}=1.1765$$

is satisfied, so the trivial equilibrium point $({S}^{0},\mathrm{0)}=\mathrm{(2},\mathrm{0)}$ is a stable nodal point. Therefore for these parameters, $x(t)$ in system (1.3) also dies out. Numerical simulation in Fig. 3 provides clear support.

**Example 5.4** We decrease the dilution rate $Q$ to 1.6, and choose the same noises densities as that in Example 5.3, then

$$1.680=Q+\frac{1}{2}{\sigma }_{2}^{2}=\tilde{Q} < m=2,\,\mathrm{(1}-4aK){\tilde{Q}}^{2}-2\tilde{Q}m+{m}^{2}=-0.5750 < 0.$$

**Figure 4:**
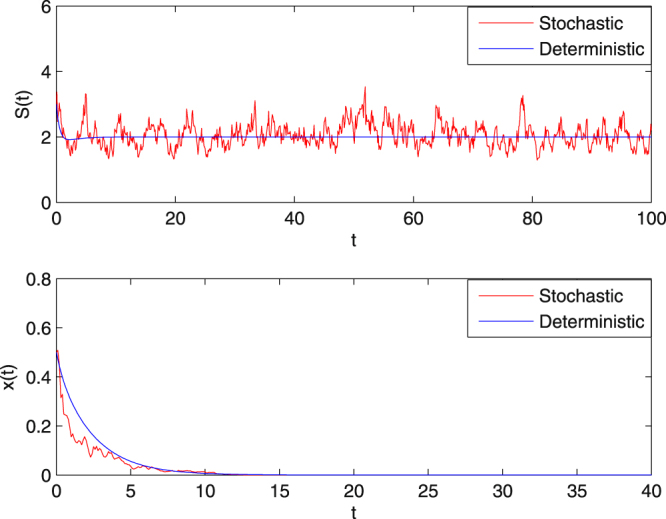
The sample paths of *S*(*t*) and *x*(*t*) for system (1.5) and the corresponding deterministic system (1.3) with *Q* = 1.6. For the intensity of the Brownian motion chosen to be ${\sigma }_{1}=0.3,\,{\sigma }_{2}=0.4$. In this case, condition (**ii**) in Theorem 4.1 is satisfied. The microorganism population dies out in both cases.

Therefore, by Theorem 4.1 (**ii**), the solution of (1.5) obeys

$$\mathop{\mathrm{lim}\,{\rm{\sup }}}\limits_{t\to \infty }\frac{1}{t}\,\mathrm{log}(x(t)) < -1.6798 < 0\,a\mathrm{.}s\mathrm{.,}$$

and so $x(t)$ tends to zero exponentially with probability one. These results are illustrated in the numerical simulations in Fig. 4. We can observe from Figs 3–4 that under the same random noises, the extinction time of the microbial population occurs later when ${Q}=1.6$ than for $Q=2.2$ for both the deterministic system (1.3) and the stochastic system (1.5).

**Example 5.5** As a comparison, we reduce the volumetric flow rate *Q* to $1.1$, and increase the intensity of Brownian motion ${B}_{2}(t)$ to ${\sigma }_{2}=1.5$ with other parameters and initial value unchanged. Condition

$$2.2250=Q+\frac{1}{2}{\sigma }_{2}^{2}=\tilde{Q} > m=2$$

of Theorem 4.1 (**i**) is satisfied, so the solution of system (1.5) obeys

$$\mathop{\mathrm{lim}\,{\rm{\sup }}}\limits_{t\to \infty }\frac{1}{t}\,\mathrm{log}(x(t)) < -\tilde{Q} < 0\,\,a\mathrm{.}s\mathrm{.,}$$

which means $x(t)$ of system (1.5) will go to extinction with probability one. Note that

$$1.1=Q < \frac{m{S}^{0}}{a+{S}^{0}+K{{S}^{0}}^{2}}=\mathrm{1.1765.}$$

**Figure 5:**
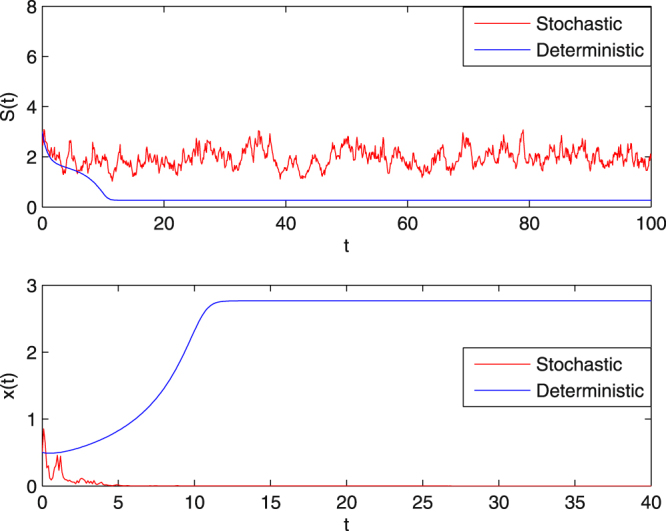
The sample paths of *S*(*t*) and *x*(*t*) for system (1.5) and its corresponding deterministic system (1.3) with *Q* = 1.1, while the intensities of Brownian motion are increased to ${\sigma }_{1}=0.3,\,{\sigma }_{2}=1.5$. The large scale of white noises cause the death for $x(t)$ in the stochastic chemostat model, while the deterministic one is survival.

In this case, the corresponding deterministic chemostat model (1.3) has only one interior positive equilibrium ${E}^{\ast }=\mathrm{(0.2715},\,\mathrm{2.7657)}$ and E* is a globally asymptotically stable node. The numercial simulations in Fig. 5 confirm the extinction of $x(t)$ in the stochastic system (1.5) and the stability of E* in the deterministic system (1.3), and it is clear that with the increasing of the intensity of white noise, the tendency for exponential extinction increases.

## Discussion and Conclusion

Understanding the effects of environmental stochasticity on the survival of microbial populations is of theoretical and practical importance in population biology. In this paper we consider a stochastic chemostat model with the Monod-Haldane response function. We first determine when this system has a unique global positive solution. Then we show that the stochastic chemostat model admits a unique stationary distribution which is ergodic if the scale of the random perturbations is relatively small. Sufficient criteria are provided for the extinction of the population of microorganisms. We also find that both strong random noise and a large dilution rate can lead to extinction. Our conclusions are all expressed in terms of the system parameters and the intensity of the Brownian motion. This means that white noise can have a major impact on the survival or extinction of microorganism populations.

How does environmental stochasticity change the predicted outcome for the microbial population in (1.3)? Actually, the stochastic chemostat (1.5) has no equilibrium. The stationary distribution shows that the solutions of the stochastic system fluctuate in a neighborhood of the positive equilibrium of the corresponding deterministic system, which can be regarded as weak stability. In Theorem 3.2, we define a new dilution rate $\tilde{Q}$ which is in terms of the original dilution rate $Q$ and the random noise on the microbial population ${\sigma }_{2}$, i.e. $\tilde{Q}=Q+\frac{1}{2}{\sigma }_{2}^{2}$. For the parameter conditions in^16^ and some restrictions on the intensity of the white noise, the stochastic system (1.3) has a unique stationary distribution and it is ergodic, which means the microbial population survives for all time regardless of the initial conditions. This result illustrates that random noise in low levels is advantageous for species survival. In Figs 1 and 2, numerical simulations show the dynamics of model (1.5) with different ${\sigma }_{i}^{2}$, $i=\mathrm{1,2}$. Comparing Fig. 1 with Fig. 2 we see that with increasing ${\sigma }_{i}^{2}$, the oscillations become stronger, but both figures reveal that the microbial population persists and demonstrate the existence of the unique stationary distribution.

With increasing *Q*, Theorem 4.1 shows that $x(t)$ tends to extinction exponentially. Furthermore, it can be observed from Figs 3–5 that the extinction of the microbial population occurs more quickly for system (1.5) with ${\sigma }_{2}=1.5$ than that with ${\sigma }_{2}=0.4$. However, with respect to the corresponding deterministic model, $x(t)$ in system (1.3) with $Q=2.2$ tends to extinction faster than that with $Q=1.6$. That is, if the volumetric flow rate *Q* is increased, the microbial population tends to extinction faster. Thus from the biological point of view, a well-controlled volumetric flow rate in the chemostat is also the key to getting a successful continuous culture of microorganisms.

We have already shown that if the densities of the environmental noise are small, the deterministic and stochastic systems have similar dynamical behavior, both in the case of persistence and extinction. Taking the dilution rate $Q=1.1$ in system (1.3) with ${\sigma }_{i}=0$, $i=1,2$, there exists an asymptotically stable interior equilibrium. Increasing ${\sigma }_{2}$, the stochastic trajectory $x(t)$ will first fluctuate around this equilibrium, then the phenomenon of noise-induced extinction occurs when ${\sigma }_{2}$ is big enough. In this stochastic case, the microorganism tends to extinction exponentially even faster than in the case of large dilution rate $Q=2.2$. These results are summarized in the following Table 1.

**Table 1:** Effects of the dilution rate and stochasticity on the survival-extinction of the microorganism.

Microbial population	*Q* ↓ (1), *σ*_2_ ↓ (0.1)	*Q* ↓ (1.1), *σ*_2_ ↑ (1.5)	*Q* ↑ (1.6), *σ*_2_ ↓ (0.4)	*Q* ↑ (2.2), *σ*_2_ ↓ (0.4)
Deterministic system	Survival	Survival	Extinction (slowest)	Extinction
Stochastic system	Survival	Extinction (fastest)	Extinction (slowest)	Extinction

**Figure 6:**
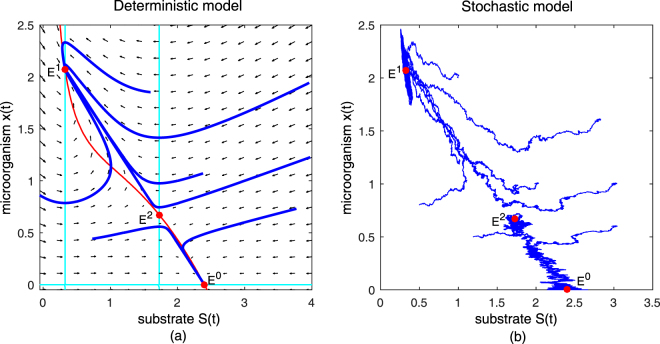
Vector field and trjactories of deterministic chemostat model (1.3) and the dynamical behavior of the stochastic chemostat model (1.5) with *Q* = 0.7. In this case, the parametric values do not satisfy the conditions in Theorem 3.2. In subgraph (**a**), both the boundary equilibrium *E* ^0^ and the positive equilibrium *E* ^1^ are locally stable nodes while *E* ^2^ is a saddle point. Choosing the intensity of Brownian motion to be ${\sigma }_{1}=0.05,\,{\sigma }_{2}=0.05$ relatively small result in small fluctuations. These solution trajectories in (**b**) have similar properties to that of the deterministic model.

To complement our theoretical result we study the global dynamics of the deterministic chemostat system (1.3) and the stochastic chemostat system (1.5) in cases that are not counter by our theorems. We set parametric values $Q=0.7,\,{S}^{0}=\mathrm{2.4,}\,m=3,\,\delta =1,\,a=\mathrm{0.9,}\,K=1.6$. In this case, $0.7=Q > \frac{m{S}^{0}}{a+{S}^{0}+K{{S}^{0}}^{2}}=0.5753$. Then according to the mathematical results in^16^, the deterministic chemostat model (3) has two interior equilibrium points, one an asymptotically stable node ${E}^{1}=\mathrm{(0.3255},\mathrm{2.0745)}$ and one a saddle point ${E}^{2}=\mathrm{(1.7281},\mathrm{0.6719)}$, as well as one boundary equilibrium ${E}^{0}=\mathrm{(2.4},\mathrm{0)}$ that is also an asymptotically stable node. The topological structure of the deterministic chemostat model (1.3) in Fig. 6(a) shows that *E* ^0^ and *E* ^1^ are both stable. The microbial population will survive if and only if the orbits tend to the interior stable node *E* ^1^. This means in order for the population to survive, we must choose appropriate initial concentrations of nutrient and microbial population. For the corresponding stochastic chemostat model (1.5), we set ${\sigma }_{1}=0.05$, ${\sigma }_{2}=0.05$. With the same initial conditions and parameter values, Fig. 6(b) suggests that processes $S(t)$, $x(t)$ move around the trajectories of the deterministic chemostat model (1.3) with small fluctuations. They have similar properties to that of the deterministic model. Note that the existence conditions for a stationary distribution are only sufficient, but not necessary. In case of $Q=0.7$, these parameters do not satisfy the hypotheses of Theorem 3.2, and so we cannot estimate whether the stochastic chemostat is ergodic or not. This is an open problem.

For biological population models, besides white noise, there is another type of environmental noise called color noise, or telegraph noise. Color noise can be illustrated as a switching between two or more environmental regimes which differ by factors such as humidity and temperature (see e.g.^32,34^). For example, the growth rates of some species in the dry season will be much different from those in the rainy season. Color noise is utilized to describe this phenomenon of environmental regimes. Color noise will disturb the steady-state of a real system indirectly by affecting system parameters such as growth rate and death rate. This is different from the perturbations of white noise that directly act on population densities (see system (1.5)). From the observation point of view, the random effect of Brownian motion is more visualized (for example, the irregular movement of pollen grains). For color noise, the switching is memoryless and the waiting time for the next switch is exponentially distributed. Hence the regime switching can be modelled by a continuous-time Markov chain $r(t)$, $t\ge 0$ with finite-state space ${\mathbb{S}}=\mathrm{\{1},2,\cdots ,n\}$. In view of the feasibility of varying the dilution rate in a chemostat (see^5^), it is also realistic to introduce color noise, or regime switching, into $Q$. Then the value of dilution rate $Q(r(t))$ will switch according to the law of Markov chain $r(t)$. For bio-mathematical model, many researchers have studied the dynamics under both white and color noise (see^27^ and the references there in). If color noise is taken into account, then we should study the ergodic property of the stochastic chemostat with Monod-Haldane growth function and regime switching. The sufficient conditions for ergodicity are supposed to be $\sum _{k\in {\mathbb{S}}}{\pi }_{k}{\sigma }_{1}(k) < \sum _{k\in {\mathbb{S}}}{\pi }_{k}{G}_{0}$, $\sum _{k\in {\mathbb{S}}}{\pi }_{k}{\sigma }_{1}^{2}(k) < -\sum _{k\in {\mathbb{S}}}{\pi }_{k}\frac{2C}{{S}^{0}}\wedge \sum _{k\in {\mathbb{S}}}{\pi }_{k}Q(k);$ and $\sum _{k\in {\mathbb{S}}}{\pi }_{k}(Q(k)+\frac{1}{2}{\sigma }_{2}^{2}(k)) < \frac{m{S}^{0}}{a+{S}^{0}+K{{S}^{0}}^{2}},$ $\sum _{k\in {\mathbb{S}}}{\pi }_{k}{\sigma }_{2}^{2}(k) < \sum _{k\in {\mathbb{S}}}{\pi }_{k}Q(k)$, which are all expressed in terms of system parameters, the intensities of Brownian motion and the distribution for Markov chain, i.e. the mathematical conclusions in Theorem 3.2 should be the mean value on space average. While in the proof of Theorem 3.2, the key is to make sure that *λ* is positive, according to condition (3.3). For stochastic system (1.5) with switching regime, it would be too difficult to find the mean value of *λ* due to the inhibitory effect. Therefore in this paper, we only introduce white noise into deterministic chemostat model (1.3).

Some other interesting topics deserve further investigation. Motivated by the work in^5,21^, one may extend our results to periodic solutions for the periodically forced stochastic chemostat model. One may concentrate on simple microbial interactions such as competitive coexistence and predation with stochasticity. We can also take other kinds of environmental noise into account, for example Lévy noise (see e.g.^35^). We leave these questions for future researches.
